# Effects of asymmetric nanostructures on the extinction difference properties of actin biomolecules and filaments

**DOI:** 10.1038/srep19658

**Published:** 2016-01-21

**Authors:** E. H. Khoo, Eunice S. P. Leong, S. J. Wu, W. K. Phua, Y. L. Hor, Y. J. Liu

**Affiliations:** 1 Institute of High Performance Computing, Agency for Science, Technology and Research (A*STAR), 1 Fusionopolis Way, #16-16 Connexis, Singapore 138632, Singapore; 2 Institute of Materials Research and Engineering, Agency for Science, Technology and Research (A*STAR), 3 Research Link, Singapore 117602, Singapore

## Abstract

In this paper, symmetric and asymmetric tapering on the arms of the gammadion nanostructure is proposed to enhance both local field distribution and extinction difference (ED). The asymmetric tapered gammadion with tapering fraction (TF) of 0.67 is seen to have the largest ED and spatial local field distribution, producing a large wavelength shift of more than 50 percent as compared to the untapered gammadion nanostructures when immersed in a solution of actin molecules and filaments. The optical chirality, ζ shows that the larger local field amplitudes produced by the asymmetric designs increases the rate of chiral molecules excitation. This enhanced field is strongly rotating and highly sensitive to single molecules and larger filaments. Here, we show that the ED, optical chirality, sensitivity and rate of chiral molecules excitation can be improved by incorporating asymmetric designs into chiral gammadion nanostructures through tapering.

Many biomolecules such as amino acids, sugars and nucleic acids are chiral. This means that their molecular structures cannot be superimposed upon their mirror images^1^,^2^,^3^. Techniques commonly used to determine optical chirality include circular dichroism (CD), optical rotatory dispersion and Raman optical activity^4^,^5^,^6^,^7^. CD is the most popular among these methods due to its rapid speed of detection, small volume of sample required and the availability of cheap equipment^5^,^6^. CD refers to the differential absorption of left- and right-circularly polarized (LCP and RCP) light and arises from the direct interaction between chiral molecules and circularly polarized light. The measured CD signal is very weak (in the range of 10^−6^ mdeg)^8^,^9^ and poses a challenge for ultrasensitive detection. Several solutions exist to enhance the CD signal for sensing chiral molecules. Fluorescent or radioactive tags attached to biomolecules^10^,^11^,^12^ have been proposed as a means to enhance the CD signal with polarized light. However, this method is not recommended due to photo-bleaching of fluorescent molecules and blocking of the active site of target chiral molecules.

To enhance the CD signal, researchers have used metallic nanoparticles to generate localized field resonance^13^,^14^,^15^,^16^. The principle behind the generation of a localized enhanced field is due to the excitation of localized surface plasmon resonances. Surface plasmons are formed by the collective free electron oscillations at metal/dielectric interfaces^17^,^18^ upon excitation by incident lightwaves. This strongly confines the light in nanoscale dimensions and greatly enhances the light field.

Nanoparticles can be placed in a helical arrangement such that they exhibit collective resonance and^16^ generate superchiral fields, prolonging and enhancing chiral interaction between molecules and lightwaves. Nanoparticles can also be assembled into a giant spiral filament via a molecular chiral scaffold^19^,^20^. These hybrid chiral materials can be tailored to be as small as clusters or as large as filaments such as DNA and protein. The nanoparticle trimer^21^, which is a composition of nanoparticles arranged asymmetrically along the planar axis, is also shown to exhibit localized optical chirality and generate superchiral near fields.

While superchiral fields can be generated by an unique arrangement of nanoparticles, the generation of superchiral fields from chiral planar plasmonic nanostructures^22^,^23^,^24^,^25^,^26^,^27^,^28^,^29^,^30^,^31^ has attracted much attention in the development of biosensors for the detection of chiral macromolecules and their hierarchical structures^29^,^32^,^33^. Chiral plasmonic nanostructures have larger surface area than nanoparticles, producing continuous superchiral fields that are of a wider coverage. The localized chiral fields interact distinctly with the accumulated molecules on the nanostructure surface. These accumulated molecules are analogous to a thin layer of dielectric material, changing the environment on the nanostructure surface and produces a shift in the CD spectrum, signalling a detection of chiral molecules. The planar gammadion is a common design for the generation of superchiral fields because it has a large dissymmetric factor^22^,^29^. Other planar chiral nanostructures include the circular disk array and G shaped structures^24^,^26^. In addition, there are also three dimensional spiral structures^34^ and multilayer planar achiral nanostructures^23^,^27^ that mimic the three dimensional properties of optical chirality. Superchirality can thus be demonstrated in many different designs of plasmonic nanostructures and metamaterials. The reported works are important scientific breakthrough for the purpose of enhancing chiral fields and serve as motivation for us to investigate factors affecting the CD spectrum of chiral plasmonics nanostructures.

In this paper, we apply tapering on the gammadion nanostructure to enhance its sensitivity. The idea of tapering arises from reported work^35^,^36^ that modifies the overall dissymmetric factor of nanostructures to increase the electric field strength for nano-focusing. We investigate the effects of tapering on the extinction difference (ED) and local field distribution through numerical simulations. Experiments on tapered gammadion nanostructures in molecular solutions are performed using broadband optical microscopy. The tapered gammadion is used to detect small chiral molecules and its associated chiral filaments. Wavelength shifts produced by different tapering designs and bio-samples of different molecular sizes are investigated to demonstrate its effects on the dissymmetric factor, sensitivity and strength of the chiral fields.

The schematic layout of a left handed plasmonic gammadion nanostructure is shown in Fig. 1a. We focus on the left handed gammadion nanostructure to demonstrate the effectiveness of different tapering methods on spatial distribution and field enhancement. The right handed structure is also expected to yield similar results^28^,^29^ [See the Supplementary Information]. The ends of the gammadion arms can be tapered via symmetric and asymmetric means. Symmetric tapering means that the width is narrowed symmetrically along the arm axis to produce an enhanced localized field^35^,^36^ as shown in Fig. 1b. Asymmetric tapering means that only one side of the gammadion arm is tapered and the distance between the arm and bend, known as the arm-bend gap remains constant as shown in Fig. 1c. By keeping the arm-bend gap constant, we can observe how field distribution between both arm and bend of the gammadion is affected when asymmetric tapering is introduced. Both symmetric and asymmetric tapering designs are adopted due to the ease of fabrication.

The width at the tapered end is defined as w_1_ and w_2_ as shown in Fig. 1b,c respectively. A metric known as tapering fraction (TF) is defined as the ratio of width, w_1_ (or w_2_) to w_0_, where w_0_ is the width of the untapered arm. TF lower or higher than 0.6 is defined as low and high TF respectively. This means that the ends of the gammadion arms have wider width for high TF. The TF of 0.6 is selected as a benchmark because it is a critical value affecting the enhanced field distribution. In this paper, we selected TFs of 0.1, 0.25, 0.5 and 0.67 to demonstrate the working principles of our design in simulations and experiments. Other TFs will also be studied to investigate the optimum TF for fabrication. Figure 1g shows the cross sectional schematic layout when the sample is immersed in water. Subsequently, the sample is immensed in G or F-actin solution when testing for biomolecules and filaments.

Figure 1d–f show the fabricated untapered, symmetric and asymmetric tapered gammadion structures for TF of 0.5 respectively. The details of the fabrication process as well as fabricated structures with TF of 0.67 and 0.25 are shown in the supplementary information. Extinction spectra was obtained using a dark field microscope with a spectrometer in the range of 400−850 nm [See the Supplementary Information]. Consisting of both absorption and scattering of light, extinction^37^ has similar line shapes as absorption, thereby allowing extinction difference (ED)^19^ to be used as a substitute for CD^38^.

ED can be defined as


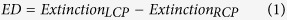


The ED spectrum for the untapered gammadion is shown in Fig. 2. We can observe three distinctive modes marked m1, m2 and m3 in the ED spectrum. Mode m1 is known as the Bloch periodic mode while modes m2 and m3 are known as the hybrid plasmonics modes^39^. Figures 3d and 4d show experimental ED spectra [See Methods] for the symmetric and asymmetric designs at different TFs respectively.

Simulations carried out for the observed experimental results are shown in Figs. 3a and 4a. The refractive indices of water and glass substrate are obtained from the Palik optical handbook^40^ while the simulated spectra is obtained by measuring the extinction of nanostructures under left and right circularly polarized light. This provides a valid and fair comparison with the experimental results obtained using the bright/dark field microscope. Figure 3a shows the simulated ED spectra for the symmetric tapered gammadion at different TFs. Figures 3b and c show the E_y_ field distributions of the symmetric tapered gammadion obtained at TFs of 0.67 and 0.1 respectively. In Fig. 3a, we observed that the simulated ED spectra exhibit similar trend as the experimental results in Fig. 3d. From Figs. 3a and d, the ED spectra for mode m1 of the symmetric gammadion are found to be weaker than the untapered gammadion. This implies that the absorption difference is weaker for the symmetric gammadion. Mode m3 red shifts to longer wavelength, while mode m2 becomes weaker and disappears as TF decreases. Finally, at TF of 0.1, only mode m1 and m3 remains. From Fig. 3c, the enhanced localized field at the lower part of the arm and its opposing bend becomes weaker and less distributed for TF of 0.1. This localized field can also be known as the gap fields. Tapering of the gammadion arms increases the gap distance between the arm and bend and makes the arm narrower. Coupling of localized fields between the arm and bend decreases for larger gap distance, resulting in weaker and less spatially distributed fields for smaller TF in mode m1. Modes m2 and m3 are due to mode hybridization^39^ in the gap between the arm and bend of the gammadion. With larger gap distance, coupling between hybridized modes weakens and mode m3 red shifts. Mode m2 become weaker and disappears at TF of 0.1.

Figure 4a shows the corresponding simulated ED spectra for the asymmetric tapered designs at different TFs. Simulations and experimental results agree very well with each other. For mode m1, ED is larger for the asymmetric tapered gammadion at TF of 0.67 and 0.5 as compared to the untapered gammadion. Figures 4b and c show the E_y_ field distributions of the asymmetric tapered gammadion obtained at different TFs for mode m1. In Fig. 4b, the local field distribution spreads over a larger part of the asymmetric tapered gammadion as compared to Fig. 3b. The gap field does not weaken because the gap distance between the arm and bend remains unchanged. Contrary to Fig. 3d, the ED spectra in Fig. 4d show stronger ED at TFs of 0.67 and 0.5 for modes m1, m2 and m3.

In order to correlate TF and the field distribution spread in ED, a parameter known as the surface enhanced field ratio (SEFR) is defined as


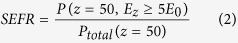


where the surface perimeter, P represents the length along the gammadion edge at z = 50 nm above the substrate with enhancement five times more than the incident field. P_total_ represents the total surface perimeter of gammadion respectively at z = 50 nm above the substrate. The parameter z is the vertical position of the gammadion. A larger SEFR indicates stronger field absorption and resonance, which in turn indicates greater circularly polarized light absorption difference or ED^15^.

Figures 5a and b show the SEFR of modes m1 and m3 for symmetric and asymmetric tapered gammadions at different TFs respectively. In Fig. 5a, it is observed that SEFR decreases after tapering is introduced for symmetric tapered gammadion. This is due to the loss of the high field region at lower TF. At TF lower than 0.6, the field at the top and bottom of the gammadion arm merges together. This resulted in weaker gap fields and smaller field distribution, indicating weaker absorption. For mode m3, the ED increases slightly before decreasing.

In the case of the asymmetric tapered gammadion, we observed that SEFR increases as TF decreases from 1 to 0.5 as shown in Fig. 5a for mode m1. This is due to the increase in amplitude and distribution of the gap fields between arm and bend for TFs ranging between 1 and 0.5. At TF lower than 0.5, the SEFR drops significantly as the loss in enhanced field region at the tapered arm is larger than the field at the arm-bend gap as shown in Fig. 4c. The results in Fig. 5b show a comparison of SEFR for mode m3 at various TFs, indicating a similar trend. In Fig. 5c, we compared SEFR at the gap for both symmetric and asymmetric tapered gammadion. For the asymmetric tapered gammadion, the field at the arm-bend gap increases as the lower part of arm has an increased field distribution and amplitude due to tapering in Fig. 4b but not Fig. 4c. Hence, tapering the arm to a smaller width (when the TF becomes small) is a disadvantage even though the field density per unit area is increased at the tapered end.

Having seen the effects of tapering on ED and the relevance of SEFR on quantifying effects of TFs on field distribution spread in ED, we investigate the sensitivity of symmetric and asymmetric tapered gammadion nanostructures on actin biomolecules. Actin^41^,^42^,^43^,^44^ is a multi-functional protein that is found in almost all eukaryotic cells. It is an important protein which takes part in many important biological processes such as muscle contraction, cell division and cytokinesis^45^. Actin exists as a free monomer is known as G-actin (globular) as shown in Fig. 6a. G-actin has a α-helix fold which is asymmetric. In the presence of ATP, G-actin can polymerize with other actin monomers to form actin filament, known as F-actin as shown in Fig. 6a. F-actin is a unique right handed dissymmetric single-stranded helix filament. The ED response of the G and F-actin are presented in the supplementary information. The biosamples are tested with both left and right handed gammadion nanostructures and the wavelength shift produced in the ED spectrum are calculated and averaged^29^. This wavelength shift is mainly attributed by the adsorption of molecules in the high field region^29^,^46^,^47^,^48^. The adsorbed G and F-actin contains tryptophan, whose carboxyl group allows binding of the actin molecules to the surface of the gammadion nanostructures and form a single flat chiral layer^46^,^49^. G and F-actin adopt geometries with a well-defined orientation axis with respect to the surface of the gammadion, and becomes randomly oriented in the plane parallel to the surface of gammadion upon adsorption.

The gammadion nanostructures of varying tapering designs are placed in a solution of different actin biosamples. The G-actin samples are prepared and stirred so that it does not polymerize to F-actin. Figure 6b shows the ED spectra for the symmetric and asymmetric tapered gammadion nanostructures immersed in a solution of G-actin molecules. We observed a larger wavelength shift for the asymmetric tapered gammadion, indicating greater sensitivity to G-actin monomers. For the symmetric tapered gammadion, the average wavelength shift is very small and is almost similar to the untapered gammadion in water. Figures 6c and d shows a comparison between the average wavelength shifts for different TFs. Mode m3 shows larger wavelength shifts than mode m1 because mode m3 is at shorter wavelength. On the molecular level, G-actin molecules have absorption energies closer to mode m3^42^. Although mode m1 has larger SEFR, it’s ED wavelength does not match the energy level of G-actin. Hence, this explains the larger wavelength shift for mode m3 compared to mode m1. We also observed that the largest wavelength shift occurs at TF of 0.67 and 0.5. As observed in Figs. 5a and b, lower TF results in smaller SEFR and hence, smaller wavelength shift. The results in Figs. 6c and d, and the SEFR results in Figs. 5a and b show a correlation between SEFR and the sensitivity of chiral biomolecules detection.

We take a solution of G-actin and add KCl to the final concentration of 100 mM. The resultant solution is left overnight to be polymerized into F-actin. To prevent F-actin from dissociating back into G-actin, we stir the solution for only 2-3 minutes to ensure homogeneity in the solution. Figures 7a and b show the average wavelength shift produced by polymerizing G-actin to F-actin. Unlike Fig. 6c, we observed a larger average wavelength shift for mode m1 in Fig. 7a. This is due to the larger size of the F-actin, which has a length of 700 nm (20 × pitch, where pitch = 35 nm). To further demonstrate the effects of F-actin on m1, we fabricated another sample with similar gammadion size and height but a period of 650 nm. With a different period, we hypothesize that the wavelength shift will be different from the gammadion sample with period of 800 nm. Figures 7c and d supports the hypothesis and show the wavelength shift produced by the symmetric tapered and asymmetric tapered gammadion for m1 and m3 at TF of 0.67. The wavelength shift reduced by approximately 12% for TF of 0.67 for mode m1. However, for mode m3, the change in period does not affect the wavelength shift much.

The above results stressed the importance of local field enhancement and its spatial distribution generated by different gammadion designs. These findings were applied to a solution of chiral molecules. However, a theoretical explanation on the improved sensitivity by using the concept of optical chirality will further demonstrate the importance of superchiral field interaction with chiral molecules. The optical chirality, proposed by Lipkin is given by^50^


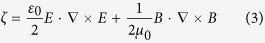


where ε_0_ and μ_0_ are the permittivity and permeability of the free space, respectively. The fields, ***E*** and ***B*** are the time-dependent electric and magnetic fields. In general, the chiral dissymmetry is proportional to optical chirality, ζ and inversely proportional to U_e_, the time-averaged electric energy^51^. This implies that chiral dissymmetry increases the rate of excitation of chiral molecules with enhanced electric field. It is deduced from Eq. 2 that SEFR is directly proportional to the rate of excitation of chiral molecules. This supports the previous results, where the enhanced field from the tapered gammadion increases the rate of chiral molecules excitation and enhanced the observed wavelength shift. This shows that tapering improves the sensitivity of the gammadion.

With incident circularly polarized light, the optical chirality, ζ can be simplified as^51^


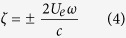


where *c* and *ω* represent the speed of light and angular velocity of rotation in free space. The “+” and “−” sign represent left and right handed circularly polarized light respectively. The time-average electric energy U_e_ can be expressed as^51^


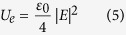


We used Eq. 5 to evaluate the optical chirality of the near field generated by the asymmetric tapered gammadion nanostructures. Figures 8a–c show the local optical chirality for asymmetric tapered gammadion at TF of 1, 0.67 and 0.1 for mode m1. We observed that the optical chirality for the asymmetric tapered gammadion at TF of 0.67 is the strongest. It’s local optical chirality spreads over a larger area compared to the untapered gammadion and asymmetric tapered gammadion at TF of 0.1. This is because tapering allows the high field region and hence the optical chiral fields to spread over a larger area as shown in Figs. 6b and c. From Fig. 8c, we also observed that the optical chirality of the asymmetric tapered gammadion is continuous as compared to the untapered gammadion. We can also relate SEFR to optical chirality, ζ. When the SEFR is large, the localized field amplitude is very strong and the distribution is large. This results in large optical chirality as ζ is directly proportional to the modulus of the electric field. A large ζ also indicates higher field rotation amplitude, as described in Eq. 2. The higher field rotation amplitude results in strong chiral field generation, creating a larger wavelength shift and improves the sensitivity of molecular detection. The optical chirality, ζ also affects the rate of excitation of chiral molecules according to the TF. As observed from Figs. 6 and 7, the rate of excitation improves for TFs ranging between 0.5 and 0.75. This range of TF produces large ζ to excite many chiral molecules, resulting in a larger change in index locally around the nanostructures. There is larger wavelength shift and improved sensitivity. A parameter called the angular velocity of rotation, *ω* is given by the equation^52^


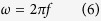


where *f* is the frequency of light. In this equation, it is observed that higher frequency results in higher angular velocity of light rotation. This rotation rate further increases the rate of excitation of the chiral molecules. Hence, we observed in Fig. 7 that the wavelength shift for mode m3 is larger as it occurred at shorter wavelength/higher frequency for G-actin molecules. However, for larger F-actin molecules, larger wavelength shift is seen for mode m1.

In conclusion, we demonstrated a new approach for enhancing ED by asymmetrically tapering the arms of the gammadion nanostructures to enhance local field strength and increase spatial distribution. Experimental results are in agreement with simulations, demonstrating that the asymmetric tapered gammadion has the highest ED and local electric field distributions at TF of 0.67. This observation is reaffirmed by the parameter, SEFR. Subsequently, the asymmetric tapered gammadion is immersed in solutions containing actin molecules and filaments. The largest average wavelength shift occurs at TF of 0.67, and it is almost double that of untapered gammadion nanostructures. We calculate the optical chirality of the tapered gammadion and explain that the asymmetric tapered gammadion nanostructures have larger spatial superchiral field distribution. The higher field enhancement can be attributed to the resulting optical chirality, ζ which increases the rate of excitation of chiral molecules and influences the rotating properties of localized field in the asymmetric gammadion structures, resulting in the larger average wavelength shift observed.

## Methods

### Experimental setup

Each fabricated sample (regardless of tapering fraction or period) has a total of approximately 4.2 ×10^7^ gammadions. The actin solution with volume of 10 μl was cast drop on top of the fabricated sample using a micro-pipette. A cover slide was then put on the top of the solution to form a uniform liquid layer with thickness of ~100 μm. All ED spectra were collected using a dark-field optical microscope (Olympus IX 71) equipped with a spectrometer (Acton SP-2357 Monochromator, Princeton Instruments). Left/Right circularly polarized light from a halogen lamp was incident from the top with the gold layer facing upward. We combined a linear polarizer and a quarter wave plate to generate either left or right circular polarization states. Light was then focused onto the substrate with the chiral nanostructures through a dry dark-field condenser and the light extinction signal was then collected with a 20× objective and directed to the spectrometer and CCD for spectra recording and image analysis respectively. ED spectra are obtained from the experiment. For accurate and meaningful comparison, we also plotted the ED obtained from simulation.

### Solution of the actin

G and F-actin solutions have a concentration of 4 mg ml^−1^. Further details of the actin preparation are given in the supplementary information.

### Plasmonics field simulations

Numerical simulations of the electromagnetic fields were performed using in-house finite-difference time-domain codes with mesh size of 2 nm. A nonuniform mesh is used because of the tapering design to ensure accurate simulation results. Permittivity for gold is taken from the Palik optical handbook^40^. Left and right circularly polarized light is normally incident onto the tapered nanostructures and monitors were placed at all faces of the simulation boundary to obtain the scattering, reflection and transmission spectra of the gammadion nanostructures. We then obtain the extinction spectra for the left (LCP) and right circularly polarized (RCP) light. The differences between the extinction spectra for the different tapered design at different tapering fractions gave the ED spectra.

## Additional Information

**How to cite this article**: Khoo, E. H. *et al.* Effects of asymmetric nanostructures on the extinction difference properties of actin biomolecules and filaments. *Sci. Rep.* **6**, 19658; doi: 10.1038/srep19658 (2016).

## Supplementary Material

Supplementary Information

## Figures and Tables

**Figure 1:**
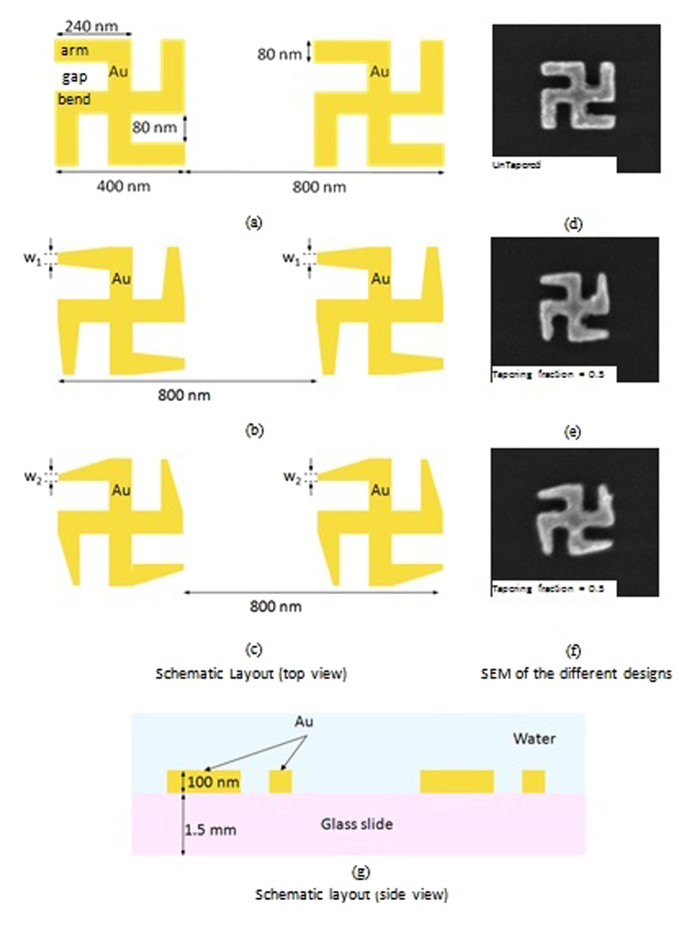
Schematic layout for designs of left handed gammadion (**a**) Untapered, (**b**) Symmetric tapered, (**c**) Asymmetric tapered. Symmetric tapering ensures symmetry along the horizontal axis while asymmetric tapering only performs tapering on one side, keeping the gap between the arm and bend constant.(**d**–**f**) Scanning electron microscopy (SEM) images of the different gammadion designs at TF of 0.5. The gammadions are fabricated using electron beam lithography and photo-resin lift-off. (**d**) Untapered gammadion, (**e**) Symmetric tapered gammadion, (**f**) Asymmetric tapered gammadion. (**g**) Cross sectional schematic layout of the gammadion designs. The gammadion is fabricated on glass slides with thickness of 1.5 mm. The SEM image of the fabricated designs at other TFs is shown in the supplementary information.

**Figure 2:**
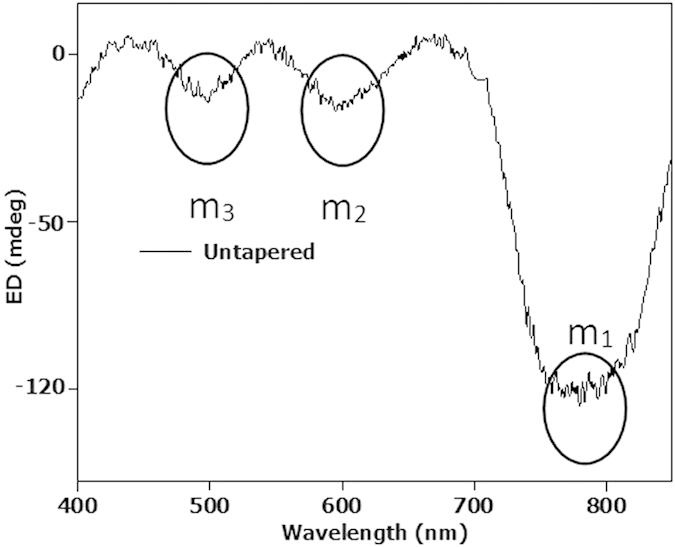
Experimental ED spectrum for untapered gammadion. We observed that there are three distinctive ED modes in the spectrum. We label these modes as m1, m2 and m3. Mode m1 is the Bloch mode due to the gammadion array, while modes m2 and m3 are hybridized modes due to coupling between the arm and bend.

**Figure 3:**
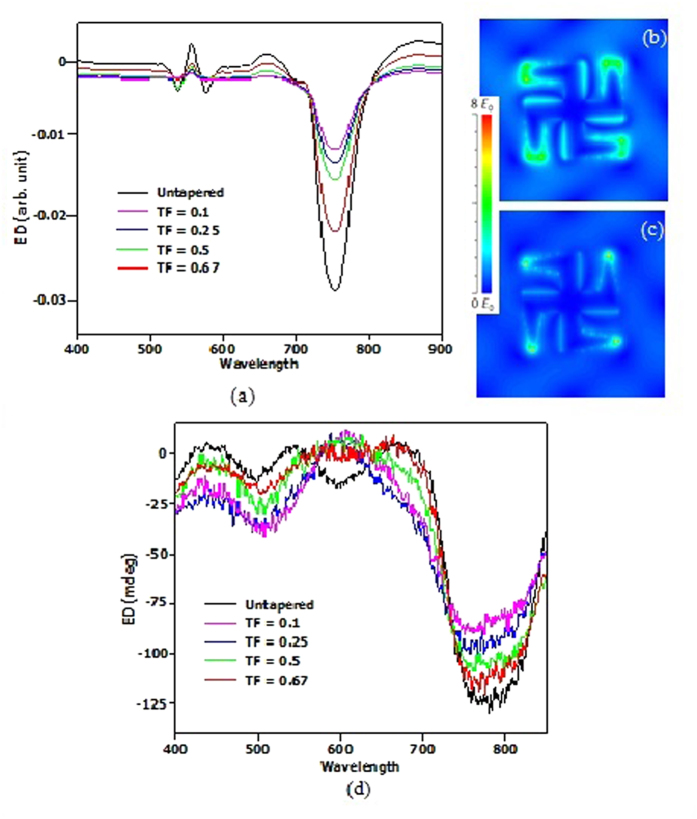
(**a**) Simulation results of the symmetric tapered gammadion. It is observed that symmetric tapering makes the ED of mode m1 weaker and causes mode m2 to disappear. The field distribution of mode m1 in the symmetric tapered gammadion is shown in (**b**,**c**), at TF of 0.67 and 0.1 respectively. From the field distribution, it is observed that the enhanced field amplitude at the end of the arm increases as TF decreases. Tapering results in an increase in field density per unit volume. However, it is also observed that the enhanced field is less distributed and only localized around the sharp edge of the symmetric tapered gammadion at TF of 0.1. The local field distribution in (**c**) becomes smaller due to tapering of the arms and increasing gap distance. Hence, we can relate the decrease in ED of mode m1 to a lesser enhanced field distribution on the surface of the gammadion. (**d**) Experimental results of the ED spectrum for the symmetric tapered gammadion. It agrees well with the simulation results shown in Fig. 3(a). The experimental results are obtained using microscopy to probe the absorption difference when incident with left and right handed circularly polarized light. The ED spectra for the symmetric tapered gammadion structures show values which are lower than the untapered gammadion nanostructures.

**Figure 4:**
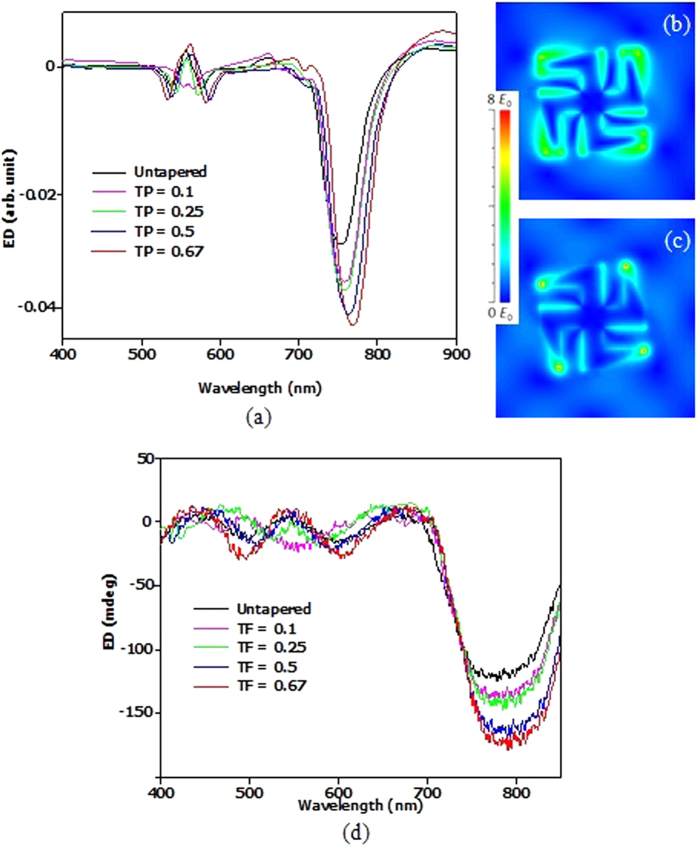
(**a**) Simulation results of the asymmetric tapered gammadion. Contrary to the symmetric tapered gammadion, the ED of mode m1 in asymmetric tapered gammadion is stronger than the untapered gammadion. The plasmonic modes m2 and m3 begin to merge as TF decreases. The field distribution of the asymmetric tapered gammadion is shown in (**b**,**c**), where the TF is 0.67 and 0.1 respectively. From the field distribution, it is observed that the field amplitude at the end of the arm increases as the TF decreases. We also observed that the enhanced field is more spatially distributed around the asymmetric tapered gammadion. This increase in field distribution occurs until TF of 0.5. The gap field also increases with tapering as shown in (**b**). (**d**) Experimental results of the ED spectrum for asymmetric tapered gammadion. It agrees well with the simulation results shown in Fig. 4(a). The experimental results are obtained using microscopy to probe the absorption difference when incident with left and right handed circularly polarized light. The asymmetric tapered gammadion shows ED improvement at TF of 0.67 and 0.5.

**Figure 5:**
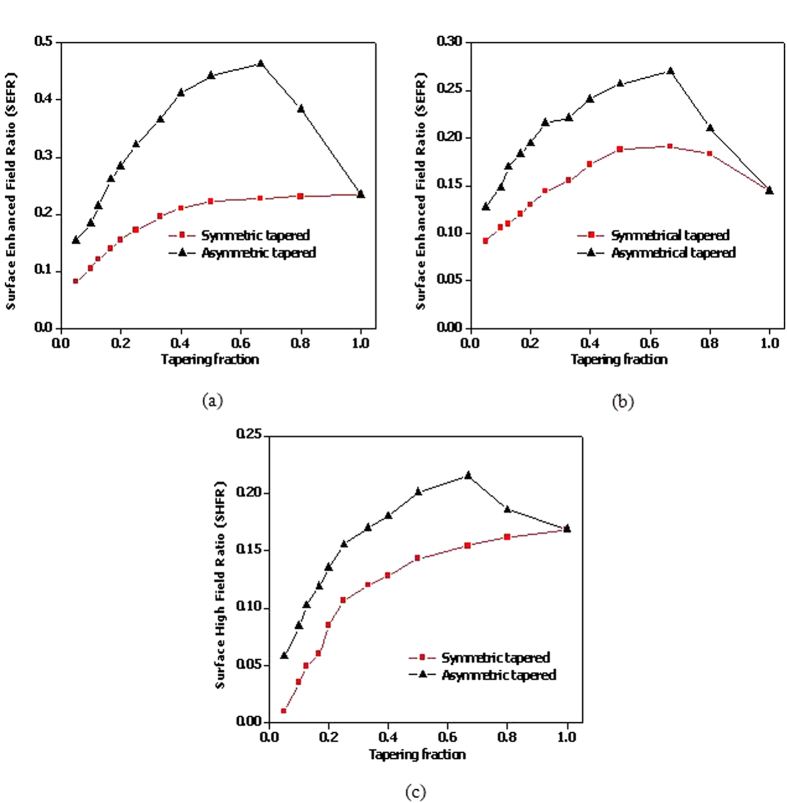
SEFR of the different gammadions for (**a**) mode m1, (**b**) mode m3 and (**c**) gap field. We observed a higher SEFR for mode m1 than mode m3. This is expected as the fields are more distributed for mode m1 than m3. It is also shown that the SEFR of asymmetric tapered gammadion is stronger than the symmetric tapered gammadion. SEFR for the symmetric tapered gammadion is seen to decrease after tapering for mode m1. For the asymmetric tapered gammadion, SEFR increases until TF of 0.67 and decreases. For mode m3, the symmetric and asymmetric tapered gammadion shows similar trend. This is because mode m3 is a plasmon resonance mode and is less affected by the initial tapering. For the gap field, it is observed that SEFR for symmetric tapered gammadion decreases from the start of tapering. For the asymmetric tapered gammadion, the SEFR increase until a TF of 0.67.

**Figure 6:**
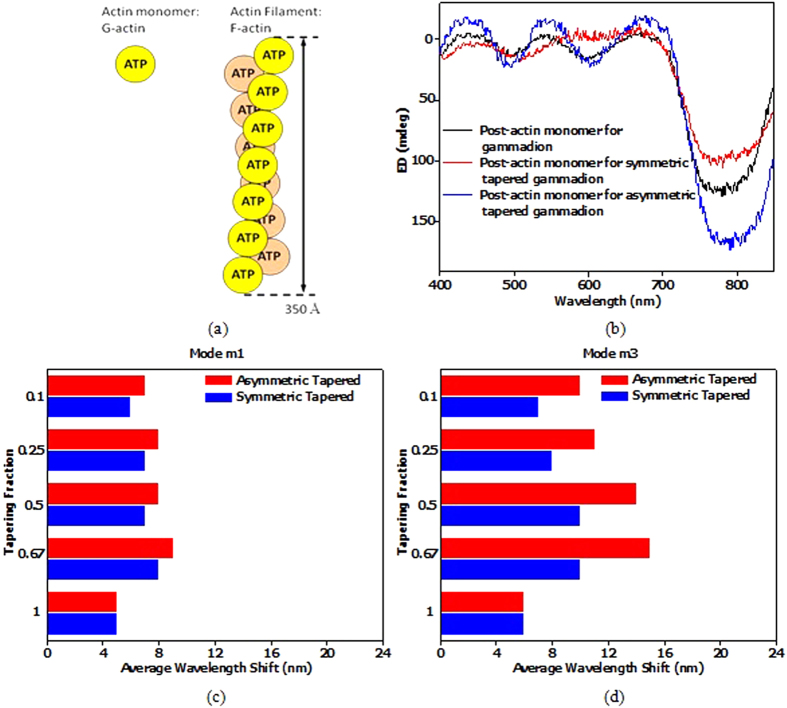
(**a**) Schematic representation of G-actin monomer and F-actin filament. The polymerization of G-actin monomer forms F-actin which is a double helix right handed filament. (**b**) ED spectra of the left handed gammadion designs after submerged in a solution of G-actin. The symmetric and asymmetric tapered gammadion designs are obtained at TF of 0.67. It is observed that the shift of mode m1 is less significant for G-actin. Mode m3′s wavelength shift is larger, indicating the properties of enhanced plasmonic mode in the gammadion. Figures (**c,d**) show the average wavelength shift for mode m1 and m3 respectively with G-actin. The average wavelength shift for mode m1 is shorter than mode m3. This is because of the different nature of the modes. Mode m1 is due to the periodicity of the array while mode m3 is due to the plasmonic resonance of the gammadion. Also, mode m3 lies at shorter wavelength and is closer to the absorption frequency of G-actin. Hence, more G-actin is absorbed for mode m3 and this explains the larger wavelength shift.

**Figure 7:**
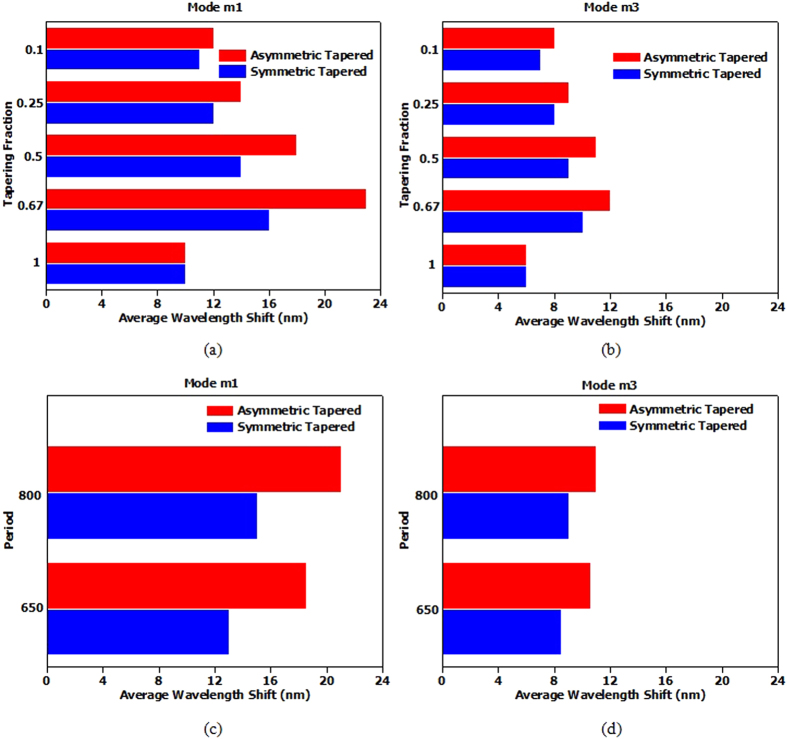
Wavelength shift of the symmetric and asymmetric tapered gammadion for modes (**a**) m1 and (**b**) m2 using F-actin filaments. We observed that the wavelength shift pattern is different from Figs. 6(c,d). Mode m1 has longer wavelength shift than m3. This is because the longer F-actin filaments are more sensitive to the periodic mode of m1. Hence, we can deduce that the longer filament is more sensitive to the ED of mode m1 at longer wavelength, while the smaller G-actin molecules are more sensitive to the ED of mode m3 at shorter wavelength. Figures 7(c,d) shows the response of F-actin on the different gammadion structures with different periods. We see that m3 is not affected much by the different periods, while mode m1′s average wavelength shift reduces when the period changed to 600 nm.

**Figure 8:**
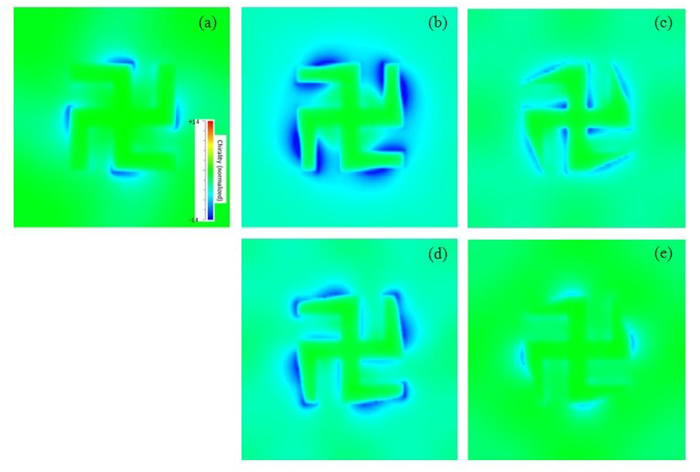
Optical chirality plots of the left handed gammadion nanostructures when incident with left circularly polarized light. (**a**) Untapered gammadion. Figures 8(b,d) show the optical chirality of the asymmetric tapered gammadion for mode m1 and m3 at TF of 0.67. Figures 8(c,e) show the optical chirality for mode m1 and m3 at TF of 0.1. We observed that the optical chirality of the asymmetric tapered gammadion at TF of 0.67 is stronger and distributed over a larger part of the gammadion. At a TF of 0.1, the optical chirality distribution is significantly smaller. For left handed gammadion, ED is negative and hence optical chirality is also negative.
