# Supplementary material for: Effects of asymmetric nanostructures on the extinction difference properties of actin biomolecules and filaments
Source: Sci Rep. 2016 Jan 21;6:19658. doi: 10.1038/srep19658 (PMC4726270; doi:10.1038/srep19658)
Supplement: Supplementary Information [file srep19658-s1.pdf]

Supplementary Information

Effects of asymmetric nanostructures on the extinction difference properties of actin biomolecules and filaments

[†1]E. H. Khoo, [2]Eunice S. P. Leong, [1]W. K. Phua, [2]S. J. Wu, [1]Y. L. Hor, [†2]Y. J. Liu

[1]*Institute of High Performance Computing, Agency for Science, Technology and Research (A*STAR), 1 Fusionopolis Way, #16-16 Connexis, Singapore 138632, Singapore.*

[2]*Institute of Materials Research and Engineering, Agency for Science, Technology and Resarch (A*STAR), 3 Research Link, Singapore 117602, Singapore.*

[†]Corresponding authors: khooeh@ihpc.a-star.edu.sg, liuy@imre.a-star.edu.sg

1. Fabrication process of the symmetric and asymmetric tapered gammadion using e-beam lithography and lift-off process

1) The gammadion designs were drawn using CAD software with a linewidth of 80 nm and a periodicity of 800 nm.

2) A 170 nm thick Poly-methyl-methacrylate (PMMA) resist was spin-coated onto a cleaned quartz substrate. The PMMA resist was baked at 180 °C for 2 min.

3) Designed patterns were exposed at a dose of 880 μC/cm^2 by using an electron beam with acceleration voltage of 100 kV and beam current of 50 pA. The exposed samples were developed in a solution of methyl isobutyl ketone (MIBK) and isopropyl alcohol (IPA) at a ratio of 1:3 for 70 s and rinsed in IPA for 20 s.

4) About 3 nm Cr was deposited followed by 100 nm Au on top of the patterned substrates by e-beam evaporation (Denton Vacuum, Explorer). The 3nm Cr served as an adhesion layer between the gold layer and glass substrate. The lift-off process was carried out by soaking in Remover 1165.

5) The samples were then rinsed with IPA and deionized water and blown dry with nitrogen gas.

6) Fabricated left handed tapered gammadion nanostructures with different tapering fractions. Figure 1a and b are symmetric tapered gammadions at TF of 0.67 and 0.25 respectively. Figure 1c and d are asymmetric tapered gammadions at TF of 0.67 and 0.25 respectively

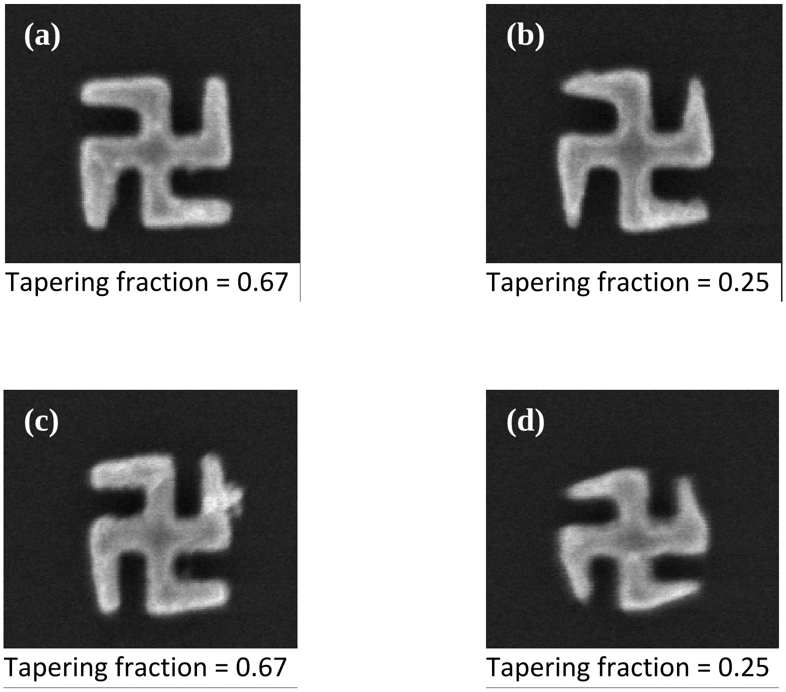

<u>2. Characterization and measurement of the symmetric and asymmetric tapered gammadion array</u>

1) An Olympus IX 71 inverted optical microscope equipped with standard dark-field (DF) condenser and spectrometer (Acton SP2300) was used for the optical extinction difference (ED) spectra measurements.

2) Light from a halogen lamp was first made either left- or right-circularly polarized (LCP or RCP) through a combination of a linear polarizer and a quarter-wave plate.

3) A dry DF condenser (NA=0.8-0.92) was used to focus the circularly polarized white light onto the sample and a 20× objective lens (NA=0.5) was employed to collect the scattering spectra and DF images through a spectrometer and CCD camera, respectively.

3. Simulation conditions for the symmetric and asymmetric tapered gammadion array

The permittivity of the glass substrate layer and gold material is taken from the Palik optical handbook in reference 40 of the main document to ensure consistency and accuracy.

1) Simulation of the double layer nanostructure is performed using finite-difference time-domain (FDTD) method with a 2nm mesh size in all directions. We use our codes written in Matlab for carrying out simulations on different gammadion design.

2) To model the system in an array, periodic boundary conditions are applied to the gammadion along the planar axis while perfectly matched layer is applied along the vertical axis of the array.

3) Left and right circularly polarized light was normally incident onto the tapered nanostructures and monitors are placed at all faces of the simulation boundary to obtain the scattering, reflection and transmission profile of the gammadion nanostructures.

4) We then obtain the extinction spectra for the left (LCP) and right circularly polarized (RCP) light. The differences between the extinction spectra for varying tapered designs at different tapering fractions give the ED spectra as shown.

5) The ED spectra for the left handed symmetric and asymmetric tapered gammadion is shown in the main document.

4. Comparison of ED and CD results

In this section, we show the simulation results for ED and CD of the left and right handed gammadion nanostructures of different tapering designs. We see that the CD and ED results agrees very well for both left and right handed gammadion with untapered and symmetric designs, especially at modes m1, m2 and m3. Similar trend is shown for the symmetric tapered gammadion, where decreasing the tapering fraction (TF) decrease the value of ED and CD. Hence, we are convinced that the results of ED can be used in place of CD in the manuscript. The CD in absorption is not exactly compensated for by a CD of the opposite sign in scattering in this design.

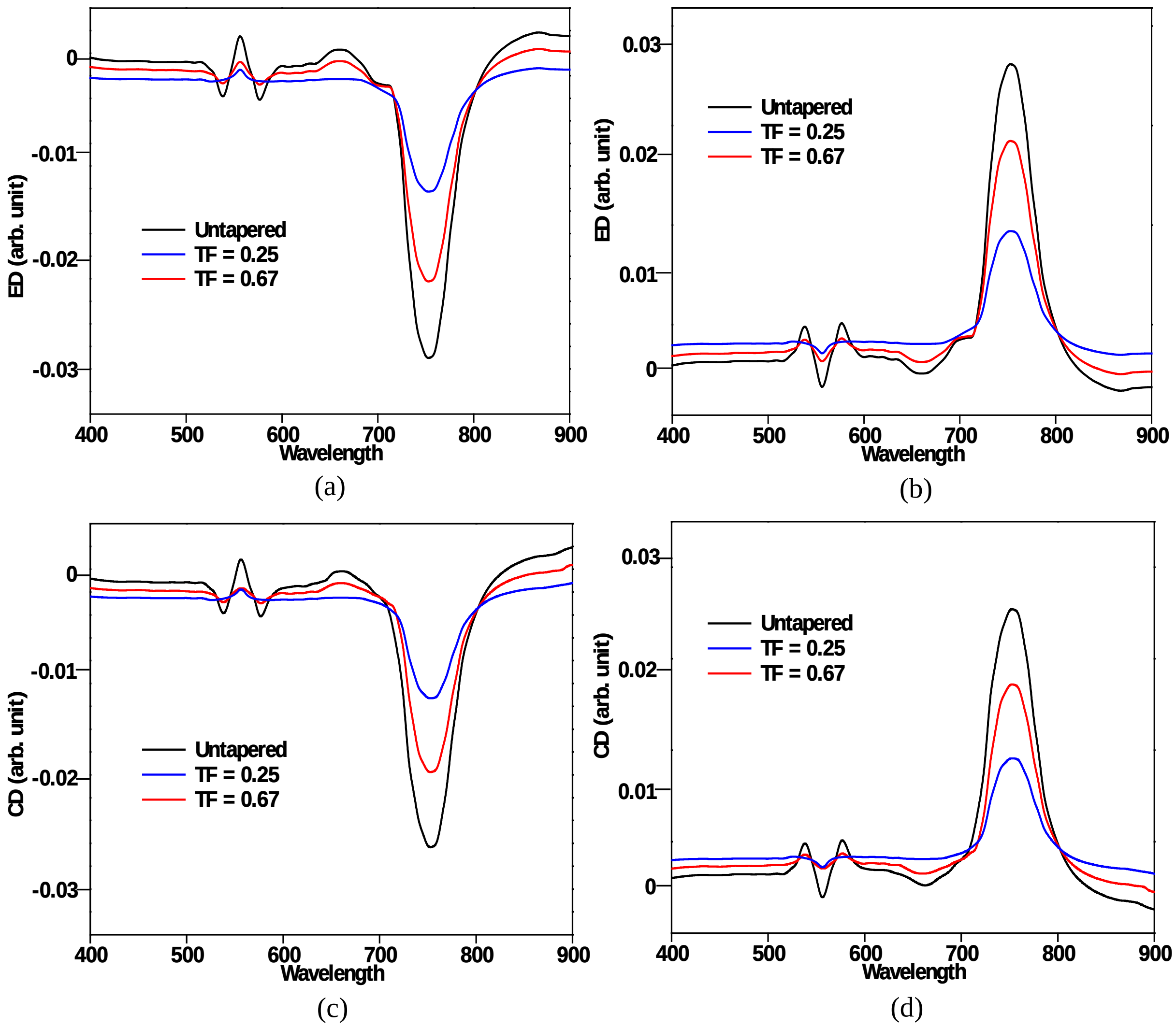

Figure 2 ED Spectra for (a) left handed and (b) right handed gammadion nanostructure. CD spectra for (c) left handed and (d) right handed gammadion nanostructure.

5. Right handed symmetric and asymmetric tapered gammadion results

In this section, the results of the right handed symmetric and asymmetric tapered gammadion are shown. The results of the left handed gammadion differs from the right handed gammadion in term of polarity; ED for modes of the left handed structure is negative and the ED for modes of the right handed structure is positive as shown in Fig. 3(a) and 3(b) respectively.

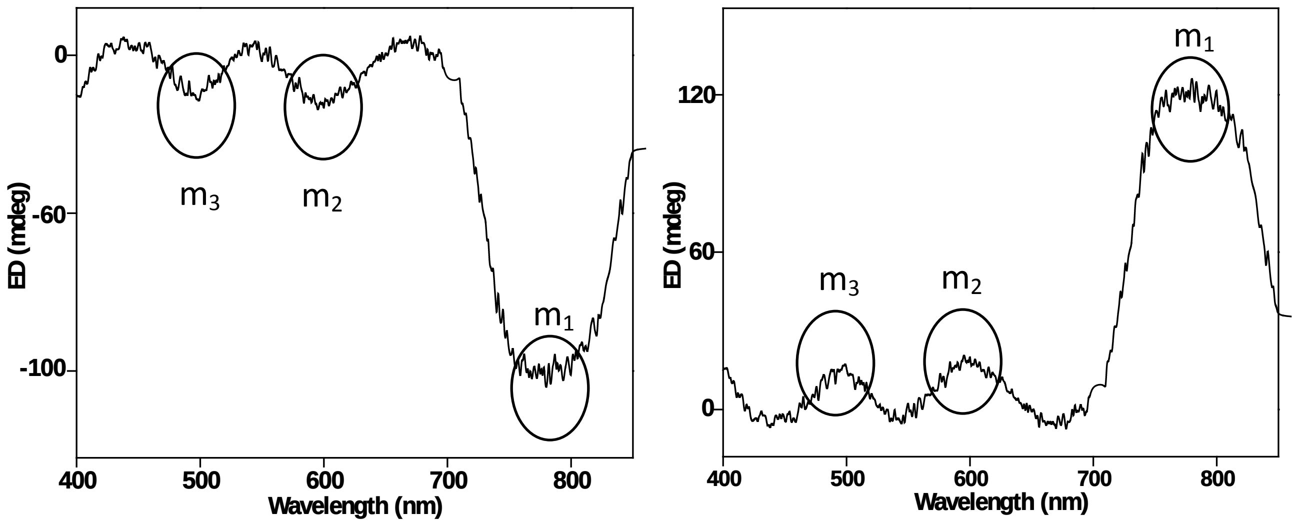

(a) (b)

Figure 3 ED experimental spectra of (a) left handed gammadion and (b) right handed gammadion.

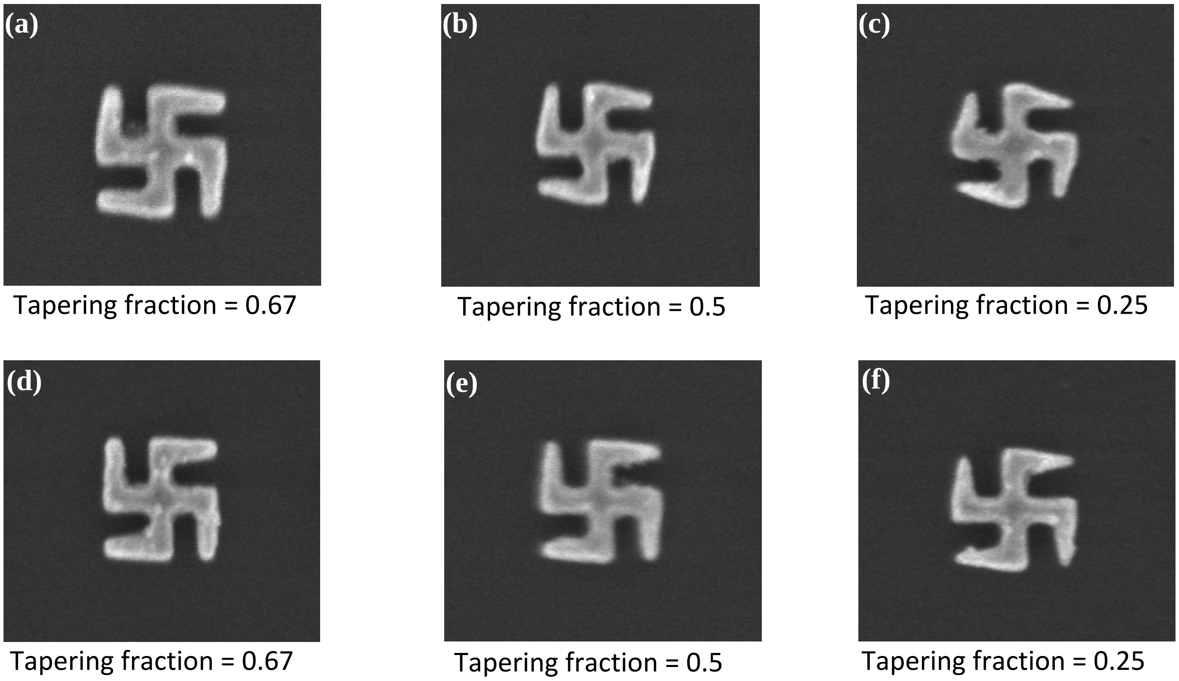

Figure 4(a)-(c) Fabricated asymmetric tapered right handed gammadion at different TFs. (d)–(f) Fabricated symmetric tapered right handed gammadion at different TFs.

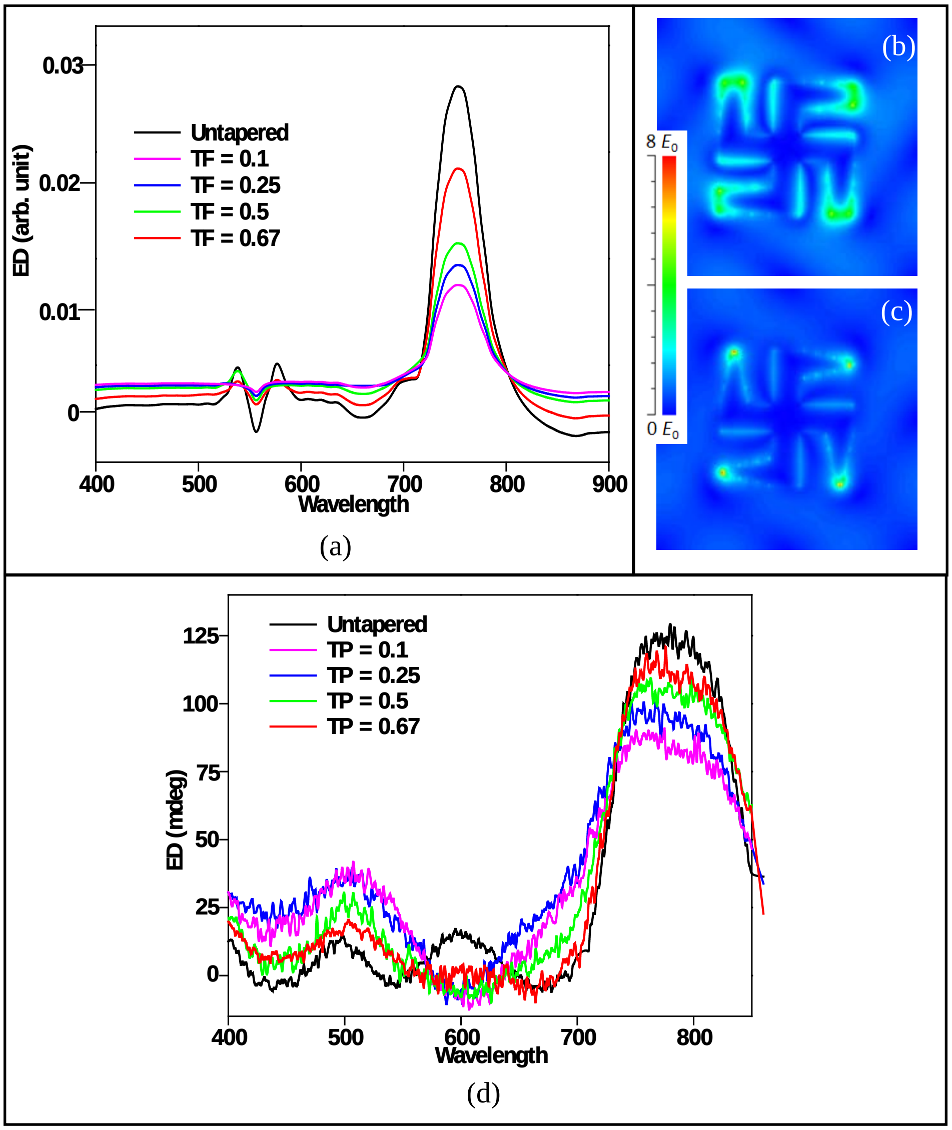

Figure 5 (a) Simulation results of the right handed symmetric tapered gammadion. The field distribution of mode m1 in the right handed symmetric tapered gammadion is shown in (b) and (c), where the TF is 0.67 and 0.1 respectively. The local field distribution in (c) becomes smaller due to the tapering of arm and an increasing gap distance. Hence, we can relate the decrease in ED of mode m1 to the lesser enhanced field distribution on the surface of the gammadion. 5(d) Experimental results of the ED spectrum for symmetric tapered gammadion. It agrees well with the simulation results shown in Fig. 5(a). The ED spectra for the symmetric tapered gammadion structures show poorer ED, with lower values than the untapered gammadion structures.

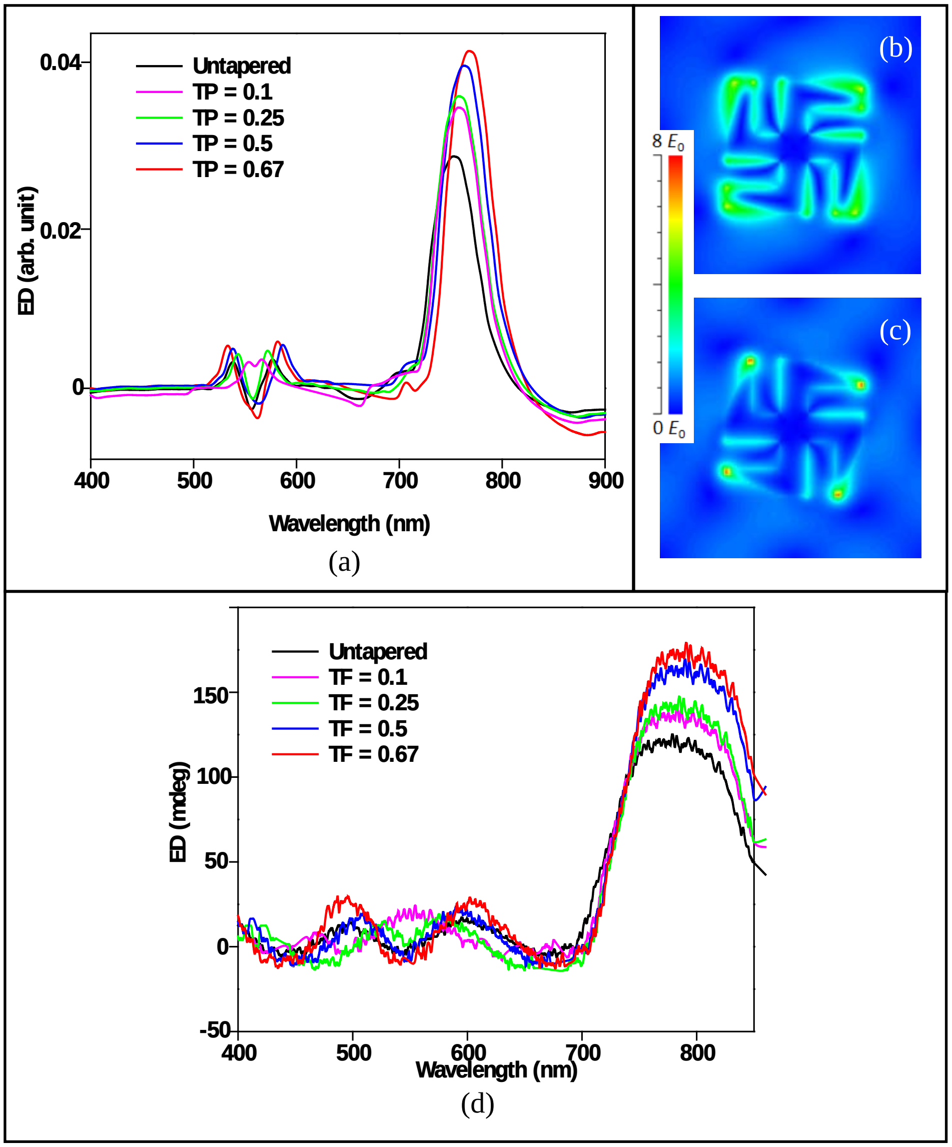

Figure 6 (a) Simulation results of the right handed asymmetric tapered gammadion. The field distribution of mode m1 in the right handed asymmetric tapered gammadion is shown in (b) and (c), where the TF is 0.67 and 0.1 respectively. Contrary to the symmetric tapered gammadion, the ED of mode m1 in asymmetric tapered gammadion is stronger than the untapered gammadion. The plasmonic modes m3 and m2 begin to merge as TF decreases. From the field distribution, it is observed that the field amplitude at the end of the arm increases as the TF decreases. 6(d) Experimental results of the ED spectrum for asymmetric tapered gammadion. It agrees well with the simulation results shown in Fig. 6(a).

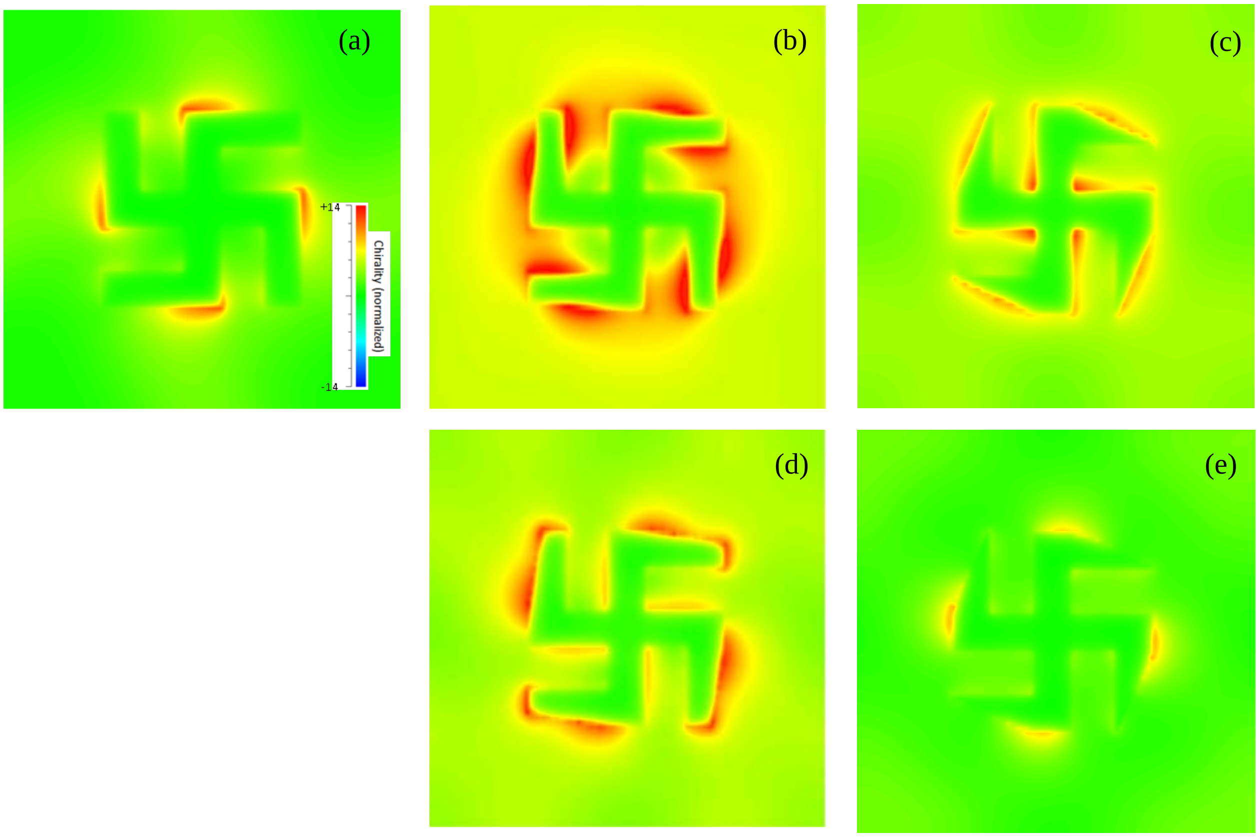

Figure 7 Optical chirality plot of the right handed gammadion nanostructures with incident right circularly polarized light. (a) Untapered gammadion. Figures 7(b) and (d) are the optical chirality plots of the asymmetric right handed tapered gammadion at mode m1 and m3 with TF of 0.67. Figures 7(c) and (e) are the optical chirality plots at mode m1 and m3 at TF of 0.1. We observed that the optical chirality of the single-sided right handed tapered gammadion at TF of 0.67 is stronger and distributes over a large part of the gammadion. At the TF of 0.1, the optical chirality distribution is significantly smaller. The chirality plot shows only positive values because the ED is positive as shown in Figs. 5 and 6 of the supplementary information.

6. Conditions for preparing G and F actin solution

5 grams of rabbit skeletal muscle powder was extracted with 60 ml of 2 mM Tris-HCl buffer, 0.2 mM $CaCl_2$, 0.2 mM Adenosine triphosphase, 1 mM dithiothreitol with buffer A at pH 7.6–7.8 for 45 min on ice. The extract was filtered through two layers of cheesecloth and filter paper, using weak suction on a Büchner funnel and flask. The residue was washed with 25 ml of buffer A and filtered again. The combined filtrates were centrifuged at 35,000 revolutions per minute (Beckman model optima L-90k ultracentrifuge and Beckman 45 Ti rotor) for 1 hour at 4°C, leaving G-actin in the supernatant.

To prepare the F-actin, the G-actin was allowed to polymerize overnight, without stirring, after addition of KCl and $MgCl_2$ to final concentrations of 100 mM and 2 mM, respectively. The concentration of G and F-actin was measured by UV-Vis spectrophotometry (PerkinElmer Lambda 4B) using an absorption coefficient of 0.63 mL/mg/cm. The concentration was 4 mg ml^{-1}.

7. ED spectra of the G-actin and F-actin

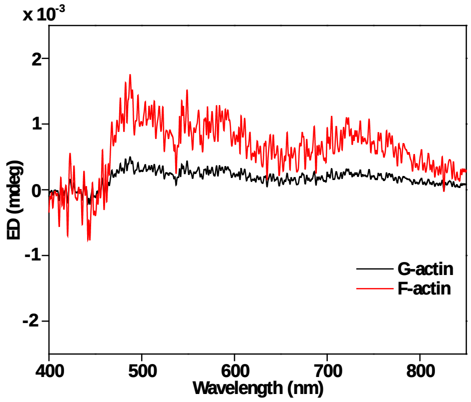

Figure 8 above shows the ED spectrum just for the molecules. The ED spectrum is obtained using the experimental setup as described in section 2 of this supplementary information. As shown in Fig. 8, the ED spectrum of the G and F-actin molecules is very small due to short absorption time with circularly polarized light.

8. Experimental ED spectra of the G and F actin molecules in left and right handed gammadion.

In this section, the experimental ED spectra measurement of the G and F-actin on the tapered left and right handed gammadion is presented. The ED of the untapered and symmetric tapered gammadion designs are given in Figs. 9(a)-(e) below.

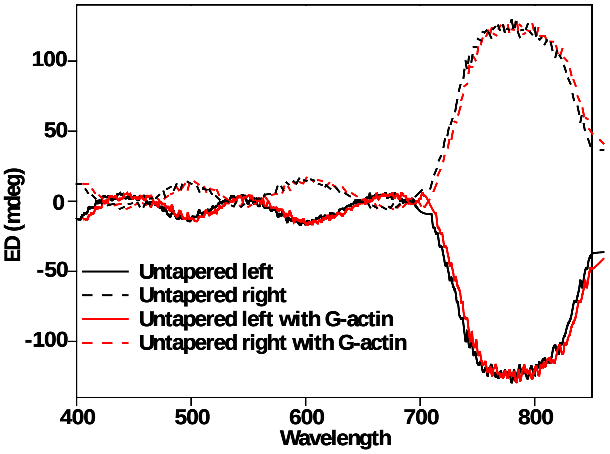

(a)

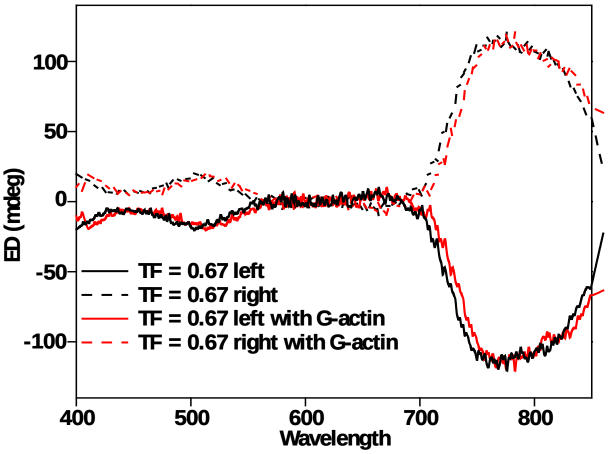

(b)

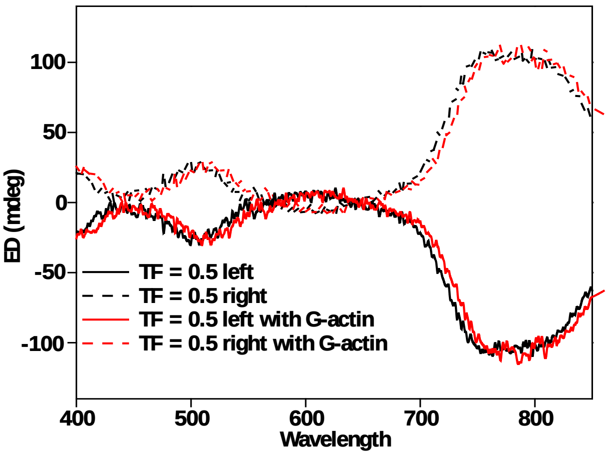

(c)

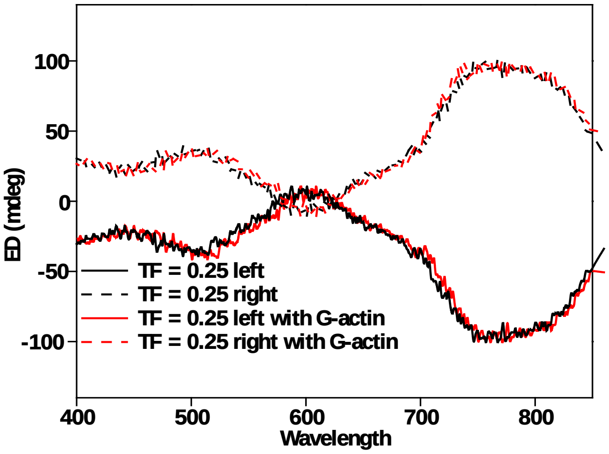

(d)

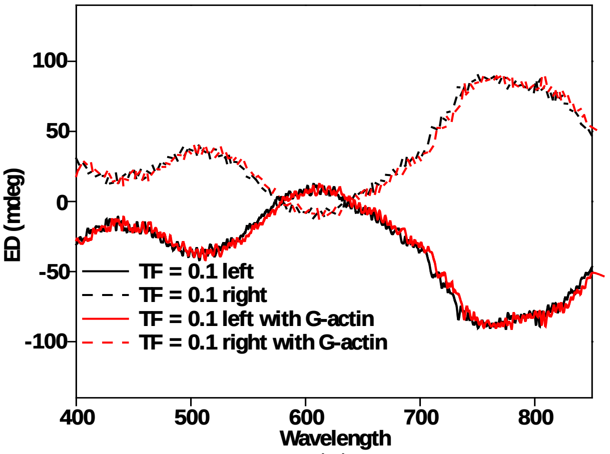

(e)

The ED of the asymmetric tapered gammadion designs are given in Figs. 10(a)-(d) below.

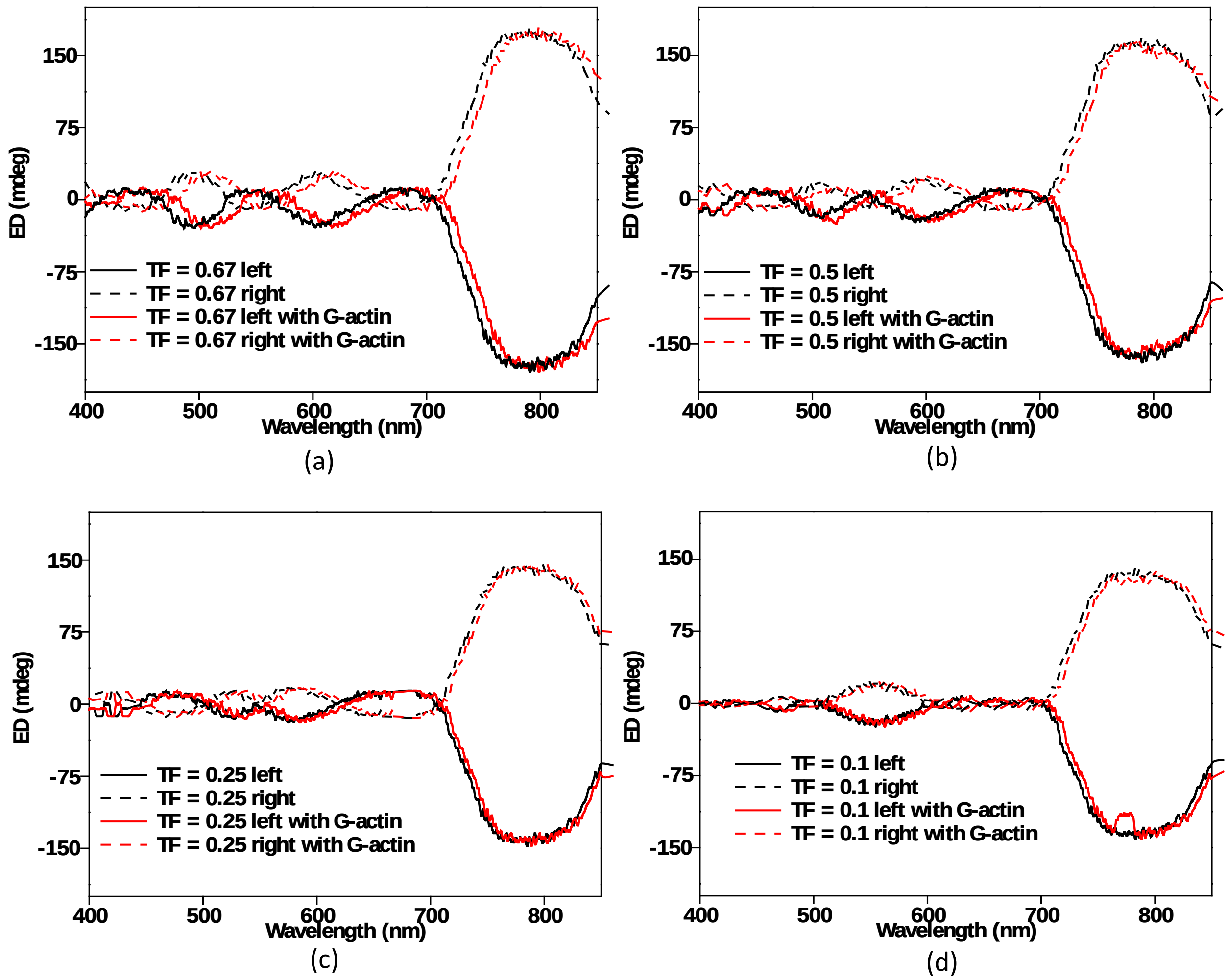

For G-actin solution, it is observed that the wavelength shift produce by mode m3 at the shorter wavelength is larger compared to mode m1. This is consistent with the results shown in the main manuscript. As explained in the main manuscript, the small size of G-actin molecules resulted in the larger wavelength shift at shorter wavelength of mode m3.

Figures 11(a)-(e) below shows the ED spectra of F-actin for symmetric tapered gammadion design. It is observed that the wavelength shift of mode m1 at longer wavelength is larger than at mode m3. This is because F-actin is a larger filament, which affects the collective formation of biomolecules on the gammadion surface.

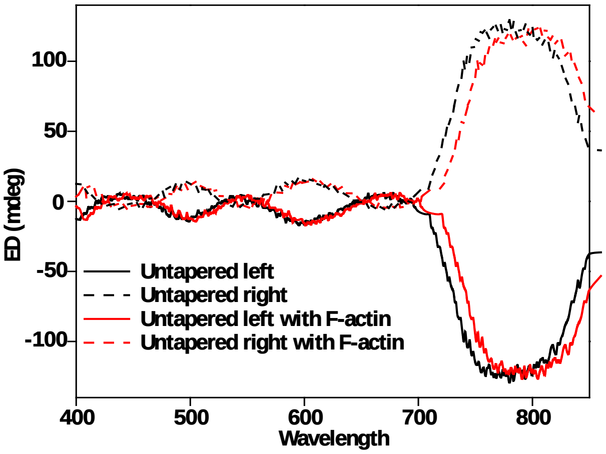

(a)

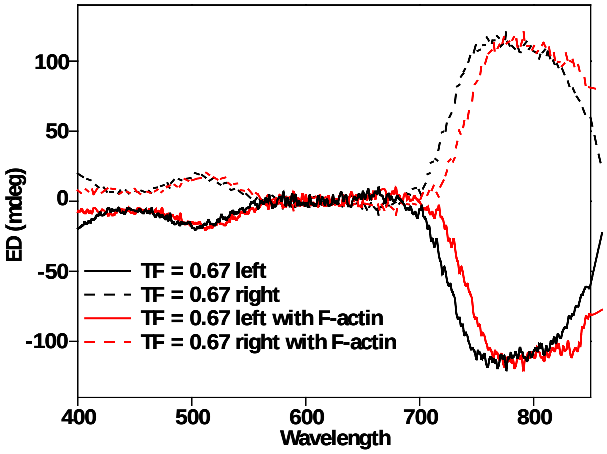

(b)

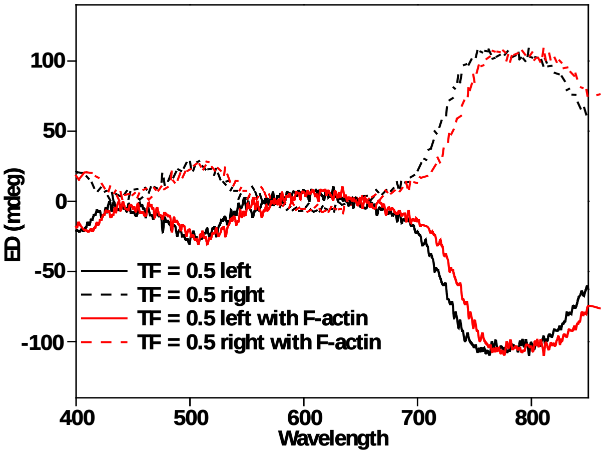

(c)

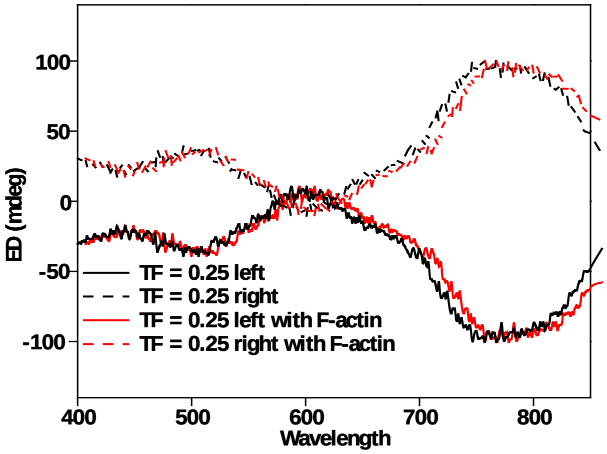

(d)

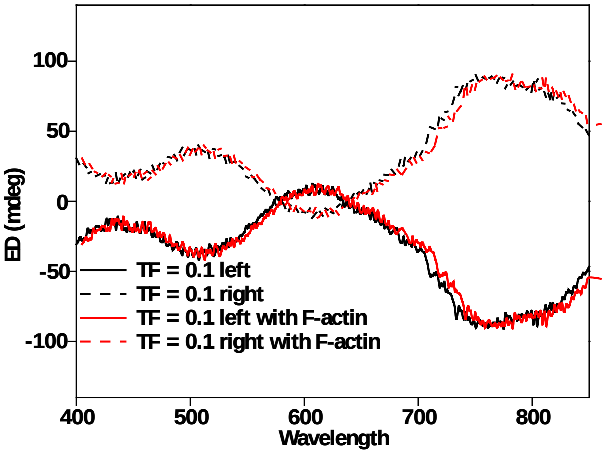

(e)

Figures 12(a)-(d) below shows the ED spectra of F-actin for asymmetric tapered gammadion design. It is observed that the wavelength shift of mode m1 at longer wavelength is larger than at mode m3. The wavelength shift for mode m1 is even larger in asymmetric gammadion design due to higher localized enhanced field.

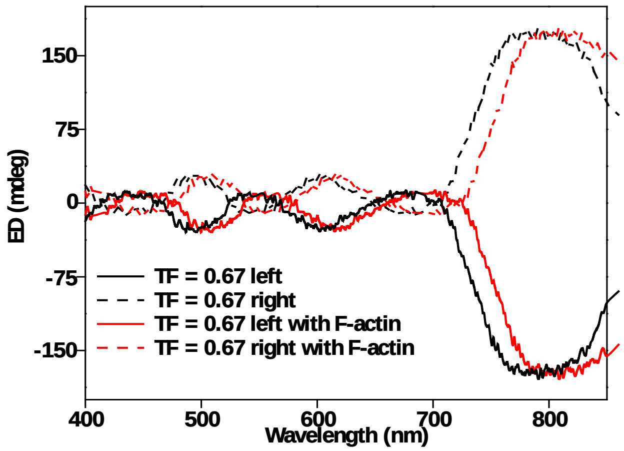

(a)

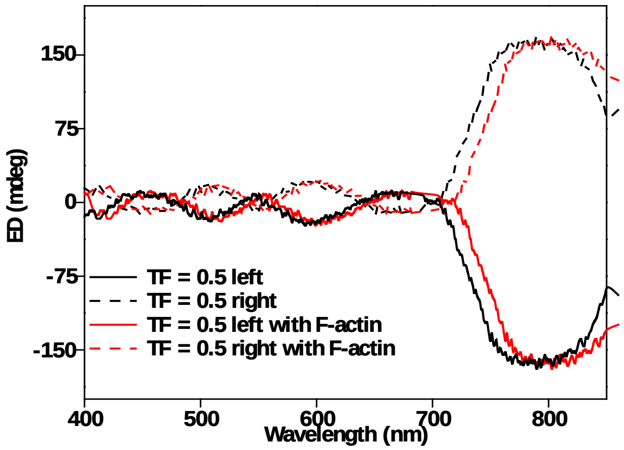

(b)

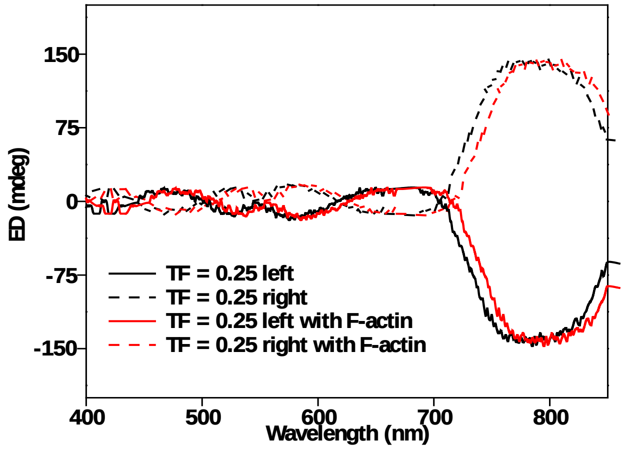

(c)

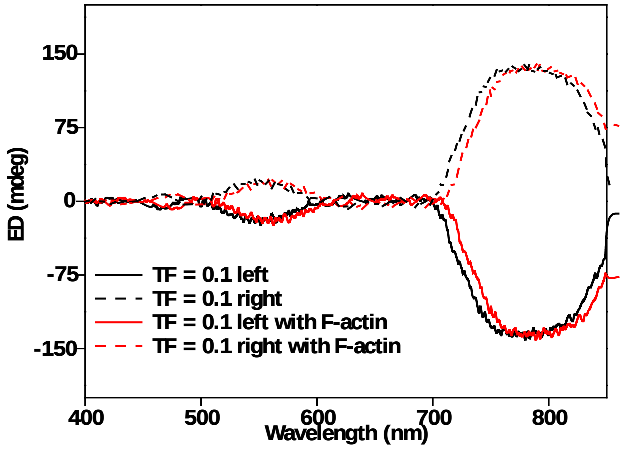

(d)

Figures 13(a) and 13(b) below shows the ED spectra in F-actin for tapered gammadion with TF = 0.67 and period at 800 nm for symmetric and asymmetric designs respectively.

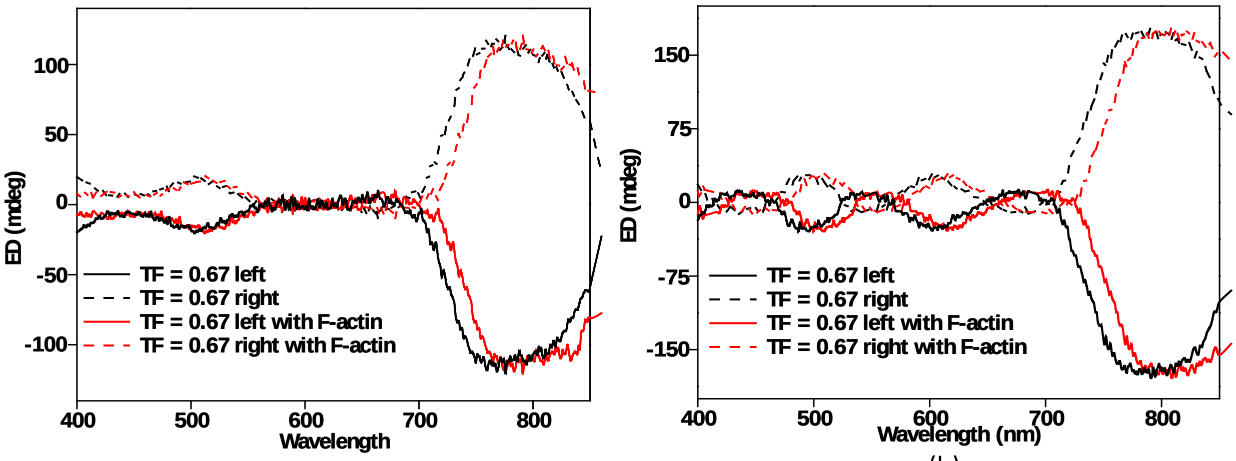

(a) (b)

Figures 14(a) and 14(b) below shows the ED spectra in F-actin for tapered gammadion with TF = 0.67 and period at 650 nm for symmetric and asymmetric designs respectively.

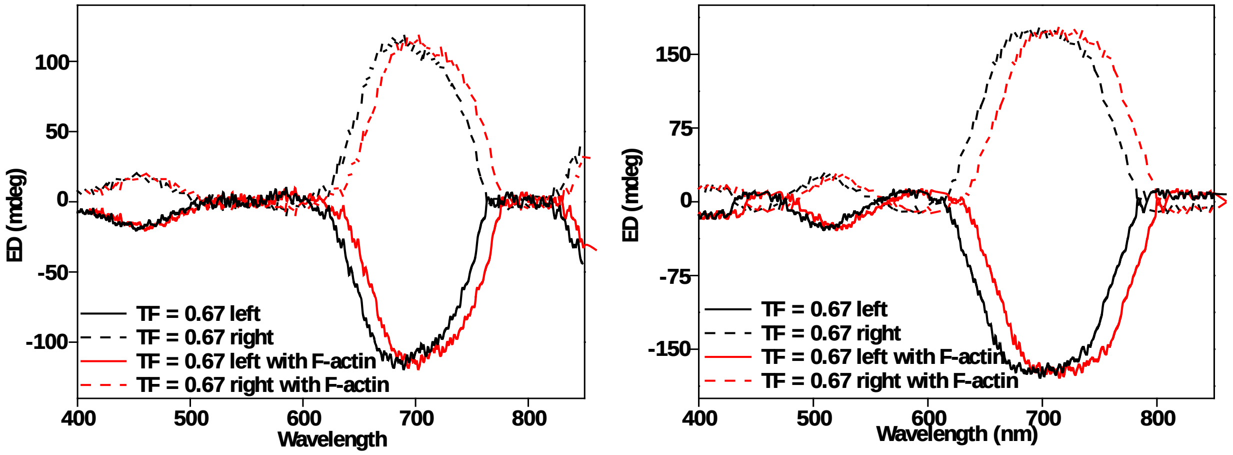

(a) (b)

It is observed from Figs. 13 and 14 that a change in period results in a larger wavelength shift on mode m1 than mode m3.
